# Cobalt Ferrite/Polyetherimide Composites as Thermally Stable Materials for Electromagnetic Interference Shielding Uses

**DOI:** 10.3390/ijms24020999

**Published:** 2023-01-05

**Authors:** Mihai Asandulesa, Corneliu Hamciuc, Aurel Pui, Constantin Virlan, Gabriela Lisa, Andreea Irina Barzic, Bogdan Oprisan

**Affiliations:** 1 “Petru Poni” Institute of Macromolecular Chemistry, 41A Grigore Ghica Voda Alley, 700487 Iasi, Romania; 2 Faculty of Chemistry, “Alexandru Ioan Cuza” University, 700506 Iasi, Romania; 3 Faculty of Chemical Engineering and Environmental Protection “Cristofor Simionescu”, “Gheorghe Asachi” Technical University of Iasi-Romania, 73 Prof. dr. doc. D. Mangeron Street, 700050 Iasi, Romania; 4 Faculty of Medicine, Discipline Biophysics and Medical Physics, “Grigore T. Popa” University of Medicine and Pharmacy, 16 University Str., 700115 Iasi, Romania

**Keywords:** polyetherimide, magnetic filler, thermal stability, broadband dielectric spectroscopy, electromagnetic shielding

## Abstract

The progress of the automated industry has introduced many benefits in our daily life, but it also produces undesired electromagnetic interference (EMI) that distresses the end-users and functionality of electronic devices. This article develops new composites based on a polyetherimide (PEI) matrix and cobalt ferrite (CoFe_2_O_4_) nanofiller (10–50 wt%) by mixing inorganic phase in the poly(amic acid) solution, followed by film casting and controlled heating, to acquire the corresponding imide structure. The composites were designed to contain both electric and magnetic dipole sources by including highly polarizable groups (phenyls, ethers, -CN) in the PEI structure and by loading this matrix with magnetic nanoparticles, respectively. The films exhibited high thermal stability, having the temperature at which decomposition begins in the interval of 450–487 °C. Magnetic analyses indicated a saturation magnetization, coercitive force, and magnetic remanence of 27.9 emu g^−1^, 705 Oe, and 9.57 emu g^−1^, respectively, for the PEI/CoFe_2_O_4_ 50 wt%. Electrical measurements evidenced an increase in the conductivity from 4.42 10^−9^ S/cm for the neat PEI to 1.70 10^−8^ S/cm for PEI/CoFe_2_O_4_ 50 wt% at 1 MHz. The subglass *γ*- and *β*-relaxations, primary relaxation, and conductivity relaxation were also examined depending on the nanofiller content. These novel composites are investigated from the point of view of their EMI shielding properties, showing that they are capable of attenuating the electric and magnetic parts of electromagnetic waves.

## 1. Introduction

Nowadays, many modern technologies, such as satellites, 5G cell phones, and radio/TV towers, operate on the basis of so-called artificial electromagnetic radiation (EMR). Such devices emit radiation in a diverse range of frequencies, which accumulate in the environment, resulting in electromagnetic interference (EMI). The latter affects the proper functionality of adjacent electronic devices by determining loss of data and signal disturbing [1]. Another undesired outcome of EMI resides in the deterioration of the health of living beings, which show serious modifications in physiological functions, genetic features, and immune system [2]. Since the exposure to EMR is unavoidable, there is an acute necessity for finding EMI shielding materials that are capable to avoid the aforementioned drawbacks. In this context, this has became a hot topic in the scientific community, which is still struggling to solve these problems [3,4,5]. Therefore, a wide variety of materials with porous/sponge [6], fibrous [7], or smooth morphology [8] were tested for EMI shielding. Their performance seems to be affected by the material composition, architecture, and the employed processing conditions.

To advance the molecular design strategies of materials, it is paramount to understand the mechanisms of EMI shielding [4,9]. The main route of EMI shielding consists of the reflection of the waves by the superficial layers of the material; this is achieved using compounds that have free charge carriers (electrons or holes). Hence, the material has to be conductive; however, raised conductivity is not specifically imposed. Due to their elevated conductivity, metals (i.e., Au, Ni, Ag, Cu, etc.) display very good shielding effectiveness (SE), but they lack flexibility, are predisposed to corrosion, and require complex processing routes [3,4]. In comparison to metals, reinforced polymers with metal or carbon fillers seem to be more advantageous as EMI shields owing to their larger flexibility, reduced costs of processing, and remarkable corrosion resistance [10]. The secondary route of EMI shielding relies on EMR absorption. This mechanism depends on the shielding material’s thickness and is also influenced by the presence of the electric and/or magnetic dipoles that are capable of interacting with the EMR [4,11]. The electric dipoles can be found in substances possessing a high magnitude of the dielectric constant (permittivity), such as SrTiO_3_, BaTiO_3_, Fe_2_O_3_, and ZrO_2_ [12,13]. On the other hand, magnetic dipoles are encountered in compounds with high permeability, such as ferrites, Fe_3_O_4_, and alloys of iron and nickel [14,15]. The efficacy of EMR absorption is augmented by increasing the frequency, thickness, and permeability of the shielding layer. When the material has no magnetic properties, EMI shielding is conditioned exclusively by the dielectric characteristics inversely [16]. Another mechanism of EMI shielding is represented by the multiple reflections, which take place when EMR meets several surfaces, phase interfaces, and in-homogeneities in the shielding layer [17]. This route is typical for materials with wide specific surface areas, but also for composites with a high-phase interface [4]. The amplitude of the EMR is considerably reduced via multiple internal reflections inside the shielding layer.

Analyzing the progress made in the area of EMI shielding materials, it seems that polymer–inorganic composites with magnetic and electric properties are the best alternative since they mingle the merits of both organic macromolecules and inorganic compounds. For instance, most polymers display mechanical resistance, flexibility in material shaping, lightweight, small thermal expansion coefficient, non-corrosiveness, and high dielectric breakdown. In order to improve their dielectric and magnetic properties, they are loaded with inorganic particles displaying larger conduction and permeability features. Regarding the typically used fillers, there is a variety of magnetic particles used for the composite preparation, such as BaTiO_3_ or Fe_3_O_4_ [18], manganese ferrite [19], magnetic Ni placed on graphene oxide layers [20], Fe_3_O_4_ or NiFe_2_O_4_/reduced graphene oxide [21,22], Fe_3_O_4_ multi-granular nanoclusters [23], and Fe_2_O_3_ combined with carbon nanostructures [24]. Among these fillers, ferrimagnetic materials, such as cobalt ferrite (CoFe_2_O_4_), are characterized by permanent magnetization, raised coercitivity, heat stability, good dielectric features, and moderate hardness [25]; however, the research regarding the implication of the CoFe_2_O_4_-loading on the physical properties of a polymer matrix is not so abundant. Landa et al. [26] introduced CoFe_2_O_4_ particles in polyaniline and observed that electrical conductivity was enhanced (reaching the metallic domain), while the remanence and coercive field were slightly diminished due to the polymer phase. Saggion et al. [27] used CoFe_2_O_4_ nanorods and incorporated them in the same conductive matrix. Their composites displayed conductivity in the semiconductor domain, whereas the magnetic features of the filler were maintained and the magnetic response of the composite was sensitive to the ferrite amount. Rashidi et al. [28] inserted this spherical-shaped filler in polyethylene glycol or polyvinyl alcohol. They noted that the second matrix was more compatible with the filler (higher dispersion), leading to reasonable magnetic properties. Mu et al. [29] prepared CoFe_2_O_4_–based composites using a fluorinated polymer as a matrix. Their materials had a magnetoelectric coefficient suitable for making AC magnetic sensors. Few studies focused on the analysis of the polymer/CoFe_2_O_4_ systems for EMI shielding. Gulzar et al. [30] embedded CoFe_2_O_4_ and fly ash into polyurethane and recorded a conductivity of around 10^−7^ S/cm, and observed a high EMI shielding of 35 dB in the interval of 0.1 to 8 GHz. Ismail et al. [31] attained polyaniline/CoFe_2_O_4_ composites and the resulting electrical and magnetic data revealed that these materials could be used as microwave absorbers (99.8% power absorption). Li et al. [32] fabricated graphite/cobalt ferrite/polyaniline systems and found that for fillers mass ratio of 1, the conductivity was around 830 S/cm, while for fillers there was a mass ratio of 0.8, where the largest magnetization was noticed. When the polymer phase was 40 wt%, the ternary composite rendered the biggest absorption of EMR of frequencies, varying between 8 and 18 GHz.

Related to the polymer matrix, polyimides (PIs) represent another category of engineering thermally stable plastics, which are largely recognized for their excellent insulation ability and high mechanical resistance [33]. PIs have the advantage that they can be shaped into membranes [34], fibers [35,36], or uniform films [37]. The aromatic structure of the PIs is beneficial for increasing the thermal resistance and electrical properties [38,39], especially when doped with adequate fillers [40,41]. The presence of ether bridges in a PI structure renders better macromolecular flexibility, facilitating the connection among the conjugated segments of the chains [42]. Hence, aromatic polyetherimides (PEI) are of particular interest since they exhibit an excellent trade-off between the processability and thermal, mechanical, and electrical properties [43,44]. PI composites with dielectric and magnetic properties have been obtained by the reinforcement with copper-nickel [45], Ba_3_Co_2_Fe_24_O_41_ [46], magnetite [47], carbon nanotubes [48], strontium ferrite [49], and other inorganic particles [50]. In any case, according to our documentation, there have not been any investigations dealing with the PEI/CoFe_2_O_4_ composites for EMI shielding applications. This represents the original point of this work, which opens new research paths toward improving the EMI shielding materials, extending their ability to withstand higher temperatures. Thus, this paper is motivated to examine the suitability of the PEI-based composites in regard to their thermal, electrical, and magnetic features and explain their involvement in EMI shielding. 

Herein, we discuss the preparation and characterization of some novel magnetic polymer nanocomposites containing various amounts of CoFe_2_O_4_ nano-sized filler. As a polymer matrix, a PEI was designed to contain aromatic rings and -CN groups considered to be sources for electric dipoles. This article evaluates the effects of nanoparticles on the thermal, magnetic, and electrical properties of the resulting films with the purpose of establishing structure–property relationships. The data collected from the magnetic and broadband dielectric spectroscopy (BDS) tests were further employed to evaluate the EMI shielding abilities of the samples. To the best of our knowledge, this is the first report on PEI/CoFe_2_O_4_ nanocomposite films, which, by means of their electrical and magnetic features, are capable of EMR attenuation via their absorbance and reflection phenomena.

## 2. Results and Discussion

The studied nanocomposites were designed based on an aromatic PEI with polar structure and ferrite nanofiller to display both electric and magnetic properties in order to be able to interact with EMR. In the next sections of the article, structural, morphological, thermal, and magnetic analyses are presented and discussed in regard to the sample composition, and EMI shielding characteristics are evaluated for the composite films. 

### 2.1. Structural and Morphological Characterization

The structure of the reference polymer film PEI-0 and of the nanocomposite films PEI-10, PEI-20, PEI-30, PEI-40, and PEI-50 was confirmed by FTIR spectroscopy. In the FTIR spectra of the samples (Figures S1–S3 from Supplementary Information), the characteristic absorption bands appeared at around 1760 cm^−1^ and 1717 cm^−1^ (asymmetrical and symmetrical stretching vibration of carbonyl groups of imide rings), 1362 cm^−1^ (C-N stretching vibration) and 742 cm^−1^ (imide ring deformation). In addition, the spectra showed absorption bands at around 3060 (aromatic C-H), 1602 cm^−1^, and 1500 cm^−1^ (aromatic -C-C-), 1240 cm^−1^ (aromatic ether linkage), and 2970 cm^−1^ and 2861 cm^−1^ (isopropylidene groups). An absorption band at 2232 cm^−1^ could be seen in all spectra due to the presence of cyano groups. The FTIR spectra of nanocomposites were almost identical to that of PEI-0, suggesting that the nanoparticles and PEI matrix interact only by physical forces and do not form chemical bonding together.

**Figure 1:**
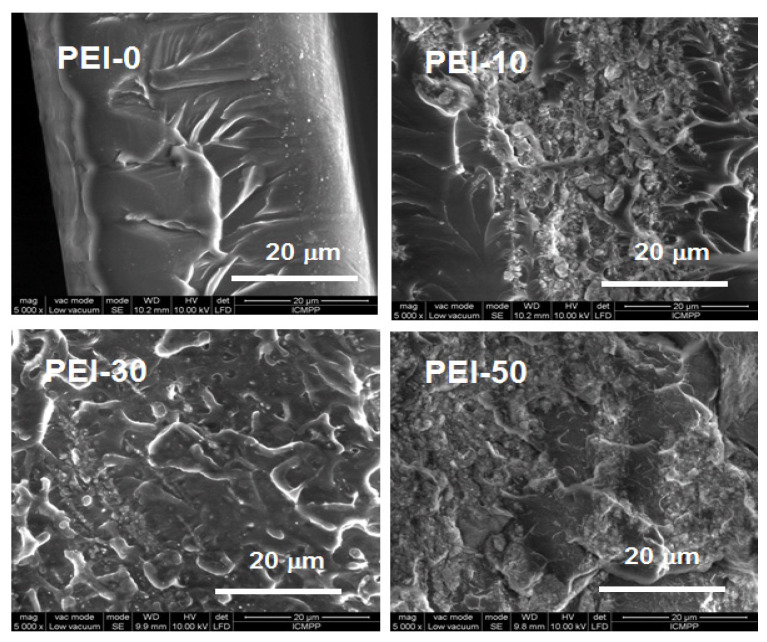
SEM micrographs of the cross-sections of the PEI/CoFe_2_O_4_ samples.

SEM was used to investigate the morphology of the neat PEI and PEI/CoFe_2_O_4_ films. The SEM micrographs of PEI-0 fractures showed low ridges and shallow cavities, indicating glassy and homogeneous microstructure. As illustrated in Figure 1, the cross-sectional surfaces of the nanocomposites were significantly rougher in comparison with the matrix PEI-0. The inserted filler was well distributed in the PEI and a low level of CoFe_2_O_4_ aggregation was noticed in small and limited regions. The dispersed particles had an irregular shape, and the few agglomerations are attributed to the magnetic dipole–dipole interactions between the inorganic nanoparticles. The surface of the nanocomposite films was not as smooth as that of the reference film since the motion of the polymer chains was restricted by the inorganic filler. In the case of the samples with high ferrite content, PEI-30 and PEI-50, it can be observed that the nanoparticles formed a continuous network. A mapping technique was used to investigate the atom distribution in the nanocomposite film. The results of the EDX mapping (Figure S4 from Supplementary Information) revealed a small level of agglomeration of the nanoparticles, especially for the samples with higher filler content. At the highest CoFe_2_O_4_ loadings in the PEI, the nanoparticles were very close to each other, forming a continuous tridimensional network. 

### 2.2. Thermal Behavior

**Figure 2:**
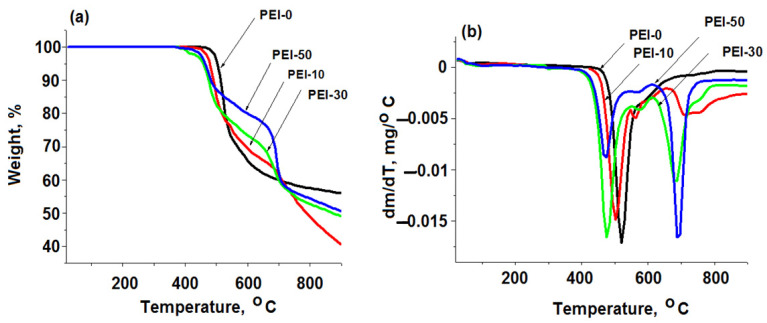
TG (**a**) and DTG (**b**) curves of the PEI-0, PEI-10, PEI-30, and PEI-50.

The thermogravimetric and thermogravimetric derivative curves (Figure 2) allow the evaluation of thermal stability. The main thermal characteristics of the neat PEI and PEI/CoFe_2_O_4_ are summarized in Table 1. The thermal stability criteria taken into consideration were the values of *T_onset_* (the temperature at which the thermal decomposition begins) and *T_peak_* (the temperature at which the degradation rate is maximum) of the first stage of sample degradation.

PEI-0 exhibits two stages of decomposition, while the samples containing magnetic nanoparticles present three stages. The thermal characteristics displayed in Table 1 reveal that the thermal stability decreased with increasing ferrite content. In addition, the mass loss percentage in the first stage of thermal decomposition decreased proportionally with the increase of ferrite content. It was established that the degradation mechanism changed at temperatures higher than 500 °C. In the case of the samples loaded with cobalt ferrite, two stages with *T_peak_* at approximately 566 °C and 689 °C were identified, compared to the neat PEI-0, which displayed one stage with *T_peak_* at 585 °C. The intermediary formed at the end of the first stage decomposed slowly in the next stage, in which the mass loss percentage also decreased with the increase in ferrite content. According to the literature [51], cobalt ferrite decomposes into metallic iron and CoO in the last stage at temperatures higher than 650 °C. This behavior has also been evidenced by the FTIR spectra recorded on residues of PEI-10 and PEI-50 heated up to 570 °C and 700 °C with a heating rate of 10 °C min^−1^ (Figures S2 and S3 from Supplementary Information). At a temperature of 570 °C, peaks at 571 cm^−1^ for PEI-10 and 584 cm^−1^ for PEI-50, which correspond to Fe-O stretching of CoFe_2_O_4_ [51], were highlighted. The peak intensity decreased in the FTIR spectra recorded at 700 °C for both samples.

The FTIR spectra recorded for residues, after PEI-0 heating to 570 °C and 700 °C, showed that at the temperature of 570 °C, it was possible to observe the characteristic bands of imide rings at 1771 cm^−1^ and those of CN groups at 2224 cm^−1^. In the case of PI-10 and PI-50, heated up to 570 °C, the characteristic bands of the imide rings and -CN groups diminished in intensity or completely disappeared, respectively, suggesting that the thermal decomposition of these groups appears easier in the presence of the cobalt ferrite, which acts as a catalyst for thermal decomposition reaction. They completely disappeared from the FTIR spectra of the residue at the temperature of 700 °C, proving that their decomposition took place in the second stage. According to previous studies [52,53] on PEI, the thermal decomposition (inert atmosphere) begins in the first stage with the breaking of the CH_3_-C link into the bisphenol A unit. 

This study also revealed that for the PEI films containing cobalt ferrite, it is possible to notice a decrease in the amount of the residue recorded at 900 °C. Similar behavior was highlighted by Martins et al. [54] for a series of poly(vinylidene fluoride)/CoFe_2_O_4_ nanocomposites. The smallest percentage of the residue was obtained for the PEI films with the lowest ferrite content. The presence of the cobalt ferrite acts as a catalyst in the thermal decomposition reaction of the neat PEI. According to the data presented in Table 1, the insertion of the CoFe_2_O_4_ in the PEI did not change the value of the glass transition temperature (*T_g_*) corresponding to the polymer matrix. The characteristics obtained from differential scanning calorimetry (DSC) curves did not change, even in the case of the study carried out by Goncalves et al. [55] for a series of poly(vinylidene fluoride)/CoFe_2_O_4_ microspheres, compared to the reference polymer. 

### 2.3. Magnetic Properties

**Figure 3:**
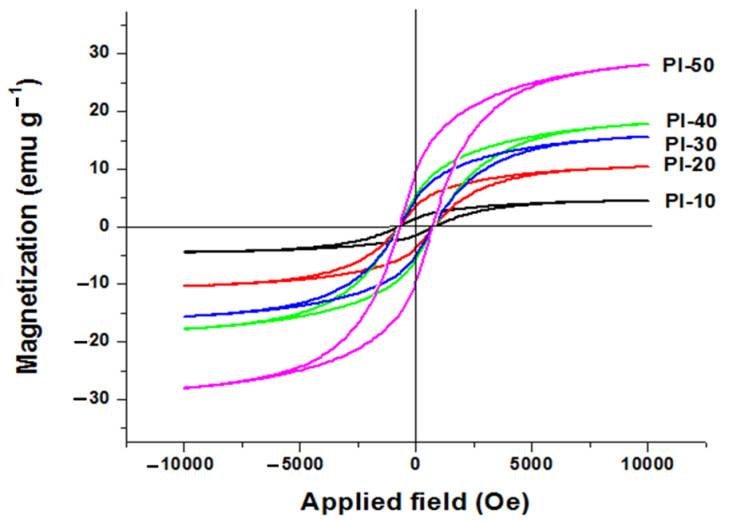
Hysteresis loops of the magnetic PEI/CoFe_2_O_4_ films.

The M-H loops for the PEI nanocomposite films having different loading of CoFe_2_O_4_ are illustrated in Figure 3. The magnetic hysteresis loop characterizes the response-ability of the magnetic material to an external magnetic field. It can provide the major magnetic parameters of the material: saturation magnetization (*Ms)*, coercitive force (*Hc*), and magnetic remanence (*Mr*) (Table 2). As expected, the neat PEI-0 did not exhibit magnetic properties. *Ms* and *Mr* increase with the ferrite content increased, the maximum values being obtained for the sample having 50% inorganic filer, PEI-50 (*Ms* = 27.9 emu g^−1^; *Mr* = 9.57 emu g^−1^). The coercivity was almost constant irrespective of the inorganic content, being close to that of the cobalt ferrite nanoparticles. The small changes in coercivity may indicate that the cobalt ferrite nanoparticles presented small variations in terms of size and agglomeration.

### 2.4. Broadband Dielectric Spectroscopy Analyses 

#### 2.4.1. The Dielectric Behavior of PEI and PEI/CoFe_2_O_4_

**Figure 4:**
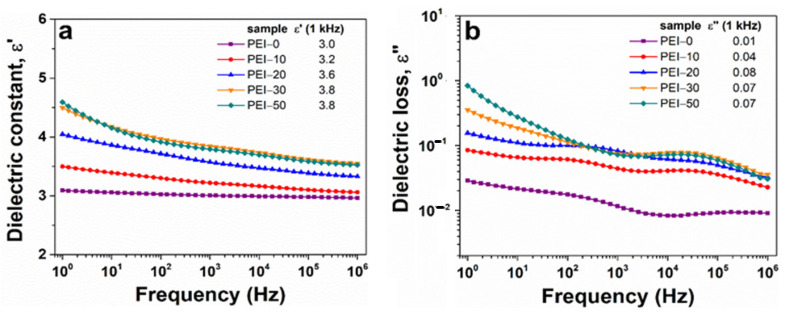
Frequency dependences of (**a**) dielectric constant and (**b**) dielectric loss at room temperature for the neat PEI-0 and the nanocomposites PEI-10, PEI-20, PEI-30, and PEI-50. The inset shows the numerical values of the *ε′* and *ε″* selected at 1 kHz.

The representative frequency evolutions of the dielectric constant (*ε′*) and dielectric loss (*ε″*) for all investigated samples are depicted in Figure 4. The dielectric constant was connected to the capacity of the chemical dipoles to align with the direction of the electrical field [56]. Thus, its magnitude was determined by the frequency of the alternating field and the temperature. According to Figure 4a, the amplitude of *ε′* was minimally reduced towards increasing frequency, especially for the simple PEI-0 sample, indicating a low dipolar activity. The latter behavior is commonly observed for aromatic PEIs [57]. The inclusion of the cobalt ferrite resulted in enhanced polarizability over the entire frequency range. The lowest dielectric constant was registered for the PEI-0 reference (inset to Figure 4a), being comparable with other PEI-type films [58], which present values between 2.8 and 3.4. The relatively low dielectric constant was due to the presence of bulky imide rings and non-polar isopropylidene units in the main chain that increased the free volume and, thus reduced the density of the polarizable units. The dielectric constant increased progressively with the addition of ferrite content in the polymer matrix. For example, *ε′* of the nanocomposite sample with 30 wt% cobalt ferrite content is enhanced up to 3.8 (*ƒ* = 1 kHz), providing an increase of 27% as compared to that of neat PEI-0. 

Following the *ε″*(*ƒ*) dependences (Figure 4b), the dipolar relaxations were detected as broad bands at intermediary and high frequencies for all analyzed samples, while the ionic relaxation could be recognized as a linear decrease of *ε″* at low frequencies, exclusively for nanocomposites with 30 wt% and 50 wt% cobalt ferrite fractions. These dielectric processes will be further discussed.

**Figure 5:**
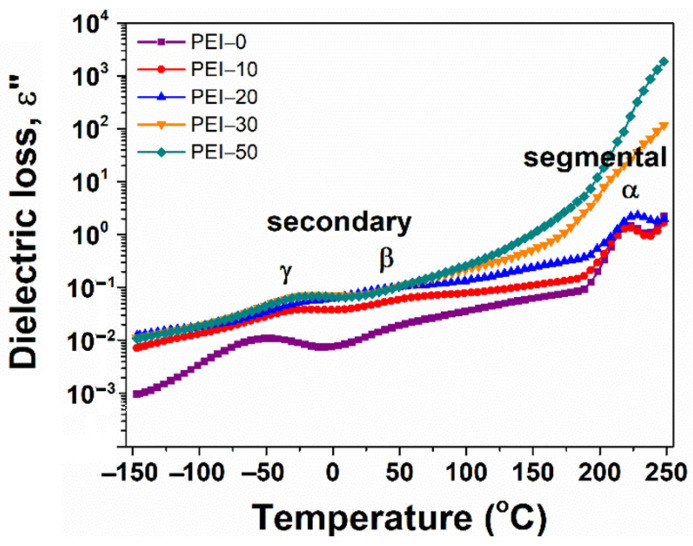
Temperature dependences of dielectric loss at 1 kHz for the simple PEI-0 and the nanocomposites PEI-10, PEI-20, PEI-30, and PEI-50.

In Figure 5, the temperature dependences of *ε″* are presented at 1 kHz for each sample. At low temperatures, the polarizable units absorbed a small quantity of thermal energy and only short segments from side-chains PEI could follow the alternating electric field. As a result, the secondary *γ* and *β*-relaxations are detected in the temperature range of −120 °C to 100 °C. The secondary γ-relaxation appeared as a dielectric band between −120 °C and −10 °C, being present in the dielectric spectra of all samples. The intensity of this transition, however, is dramatically reduced in the case of nanocomposites. According to the literature [57], the dipolar *γ*-relaxation is commonly associated with isolated motions of the small chemical species that have absorbed water from the atmosphere. At higher temperatures, the *β*-relaxation manifested as a broad band that may be assigned with short segmental motions from the main chain, including localized fluctuations of the diamine units [57,59,60]. Interestingly, the dipolar *β*-relaxation was not visible in the dielectric spectra of the PEI-0 matrix, providing that the dipolar motions associated with this transition were influenced and highlighted by the presence of the inorganic ferrite particles. The secondary relaxations’ temperature region was accompanied by a moderate increase of *ε′* with temperature, providing significant thermal stability of the prepared materials (see Figure S5 in the Supplementary Information File). With further increase of the temperature, chemical dipoles absorb a significant amount of thermal energy and, accordingly, the cooperative motions of the larger segments from the polymer backbone are activated. At temperatures around the sample’s *T_g_*, the primary *α*-relaxation is revealed as one intense peak (e.g., for the PEI-10, *α*-relaxation occurs around 210 °C) that is commonly attributed to the large-scale cooperative motions of the macromolecular chains of the PEI [61]. From the *ε″*(T) dependences for the PEI-30 and PEI-50, it is clear that the relaxation peak of the *β*-relaxation was overlapped with the conductivity of the free charge carriers. The dielectric relaxations could be better visualized on the 3D representation of the dielectric loss as function of frequency in the overall temperature range, selected for the PEI-20 sample (see Figure S6 in the Supplementary Material). 

#### 2.4.2. Segmental *α*-Relaxation Closed Connected to Glass Transition

**Figure 6:**
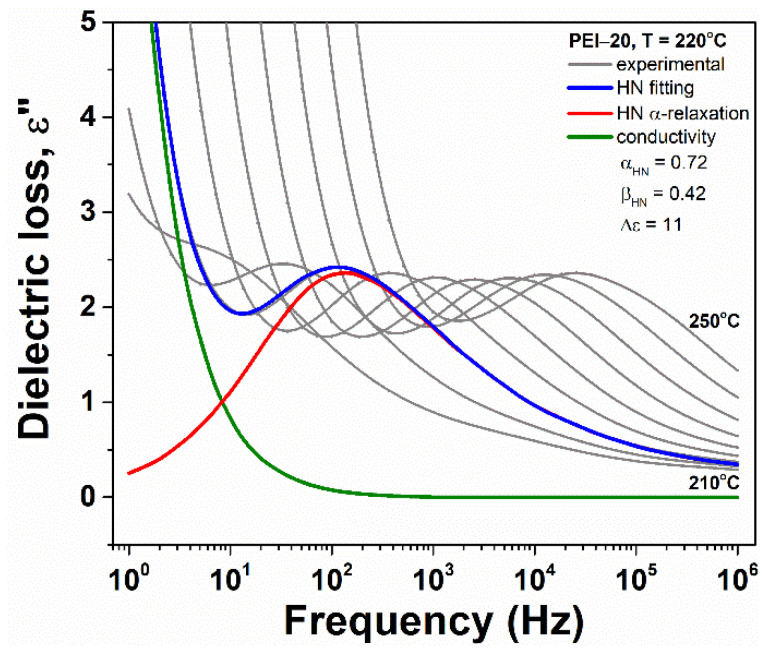
Exemplary frequency dependences of the dielectric loss at temperatures between 210 °C and 250 °C for the PEI-20 nanocomposite. A representative separation of the segmental *α* in terms of the HN fit function is provided at 220 °C. The corresponding fitting parameters are presented in the box.

The dipolar bands associated with the secondary *γ* and *β*-relaxations were broad and unclear, as previously observed. As a matter of fact, the subglass secondary relaxations could not be processed properly. Therefore, this study was focused on the evaluation of the segmental *α*, in connection with the glass transition of the bulk polymer. This dipolar relaxation was detected for the PEI matrix as well as nanocomposites containing 10 wt% and 20 wt% (Figure 5). Figure 6 shows a detailed representation of the isothermal regime of the cooperative segmental *α*-relaxation processes for the PEI-20 nanocomposite, as a representative example. The other samples revealed similar information. The transition was displayed as a broad peak superimposed with the conductivity signal and moved to higher frequencies towards the temperature, as a classic behavior of thermally activated processes. An exemplary separation of isothermal α-relaxation is also depicted in Figure 6 at a temperature of 220 °C using the Havriliak–Negami (HN) function (Equation (1)—see below Experimental section). The fit function (shown by a blue line) overlapped with the experimental data points and encompassed an HN term around 130 Hz attributed to segmental *α* (represented with a red line) and a supplementary term for the conductivity signal (represented with a green line).

**Figure 7:**
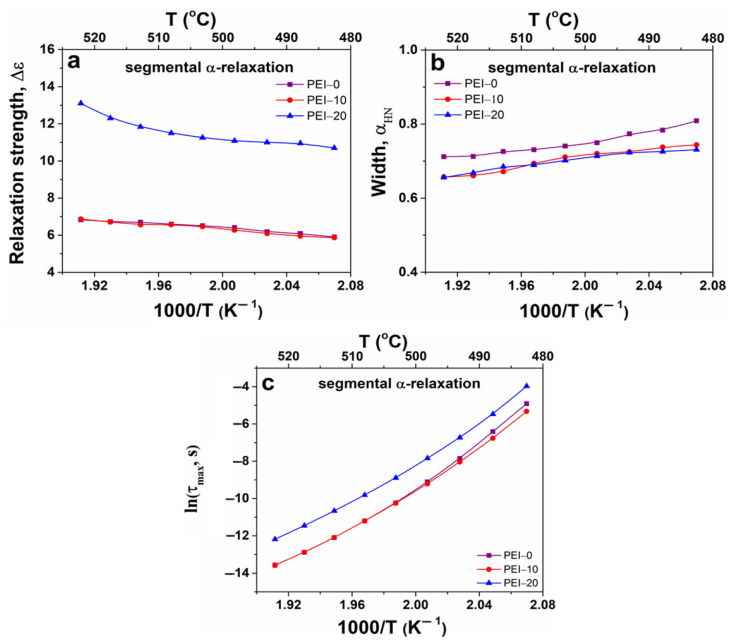
Reciprocal temperature dependences of the relaxation strength (**a**), width (**b**), ln(*τ_max_*) (**c**) parameter for the VFT fitted α-relaxation of the neat PEI and the nanocomposites with 10 wt% and 20 wt% cobalt ferrite fractions.

The evolution of the relaxation strength and width, as retrieved with the HN Equation (1), are further represented against reciprocal temperature in Figure 7a. With α_HN_ values between 0.6 and 0.8, the segmental α was characterized by the relatively narrow distribution of relaxation times. Additionally, the width parameter became larger with the incorporation of the inorganic cobalt ferrite nanoparticles and the increase in the temperature. Furthermore, the *β_HN_* parameter (not shown here) was found to be ~0.42 for all samples, providing the asymmetry of the primary relaxation. On the other hand, the intensity of the segmental *α* (the relaxation strength, Δ*ε*) is remarkably enhanced for the PEI-20 sample, highlighting that cobalt ferrite has a major impact on the dielectric properties of the corresponding nanocomposites.

The evolution of the *τ_max_* (Equation (2)—see Experimental part) as a function of inverse temperature is plotted in Figure 7b, and the numerical values of the Vogel–Fulcher–Tammann (VFT) Equation (3) (see Experimental section) are presented in Table 3.

When the inorganic nanoparticles were added to the PEI matrix, the apparent activation energy was enhanced, announcing that the segmental motions were restricted. At the same time, the Vogel temperature was steadily reduced, suggesting a decrease of the cooperativity for the segmental movements. These results allow the prediction that the incorporation of this filler would result in strong polymer/filler interaction.

#### 2.4.3. Conductivity

**Figure 8:**
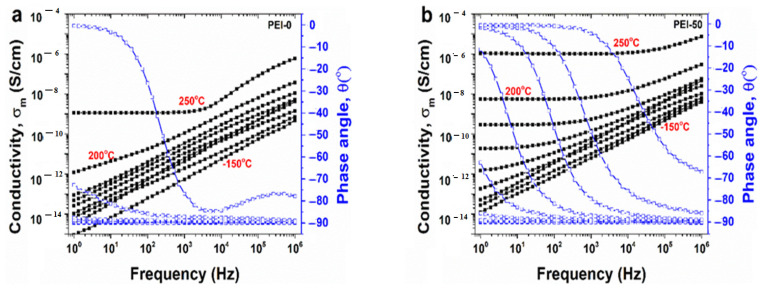
Evolution of the measured conductivity (left axis) and phase angle (right axis) as a function of frequency at various temperatures for the neat PEI-0 (**a**) and PEI-50 (**b**).

The frequency dependences of the measured conductivity, *σ_m_* (S/cm), and phase angle, *θ* (°), at various temperatures are represented in a common diagram in Figure 8 for simple PEI matrix and the nanocomposite with 50 wt% cobalt ferrite fraction, as representative examples. At low temperatures, the magnitude of the *σ_m_* increased linearly with frequency and *θ* presented values close to −90°. This capacitive behavior was governed by the dipolar relaxation processes from the polymer backbone. As the temperature increased, the measured conductivity deviated from linear tendency and the values of the phase angle were progressively enhanced up to 0°. With the further increase of the temperature, a flat plateau region of conductivity was promoted at low frequencies. This resistive behavior could be assigned to the free charge carriers moving through the polymer lattice [62]. For neat PEI (Figure 8a), the resistive behavior was developed at temperatures above *T_g_*. In the case of the PEI-50 nanocomposite (Figure 8b), the plateau region of the conductivity rose at temperatures much lower than the glassy state (e.g., for the PEI-50, the resistive behavior rises around 0 °C) because of the cobalt ferrite that acted as a source of free charge carriers. 

**Figure 9:**
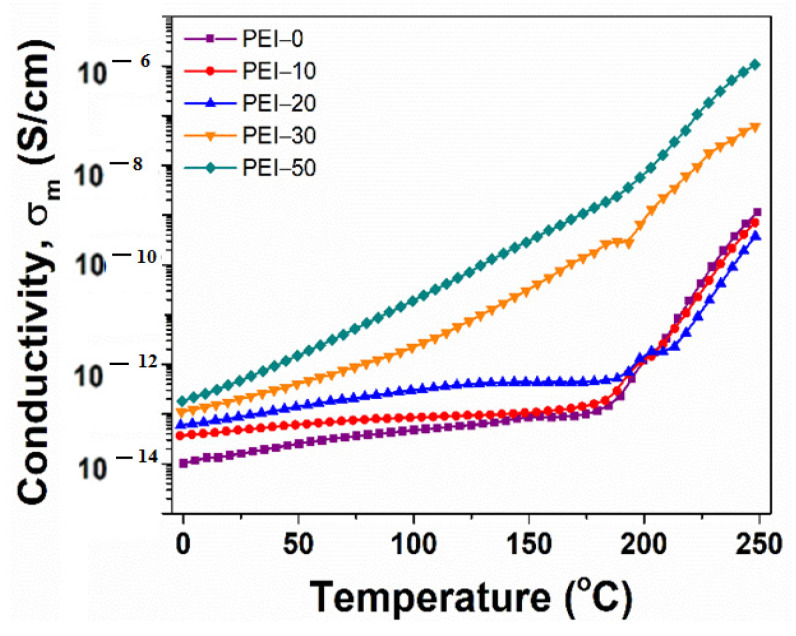
Temperature dependences of the measured conductivity at 1 Hz for the simple PEI-0 and the nanocomposites PEI-10, PEI-20, PEI-30, and PEI-50.

The influence of the cobalt ferrite content on the conductivity of the PEI/CoFe_2_O_4_ nanocomposites can be visualized in Figure 9 and Table 4.

The magnitude of the *σ_m_* of the neat polymer increased slowly with temperature up to the glassy state. Then, the conductivity was thermally activated, as generally observed for a typical PEI material [57]. The temperature behavior of the *σ_m_* for the PEI-10 and PEI-20 samples followed the tendency of neat PEI-sample, revealing the low impact of the inorganic particles. On the other hand, for the nanocomposites containing 30% and 50% cobalt ferrite, the conductivity was enhanced by at least one order of magnitude even at low temperatures, and it improved when increasing the temperature. Further magnification of the conductivity could be achieved by the incorporation of semiconductive Janus nanoparticles in the present polymer systems.

### 2.5. EMI Shielding

**Figure 10:**
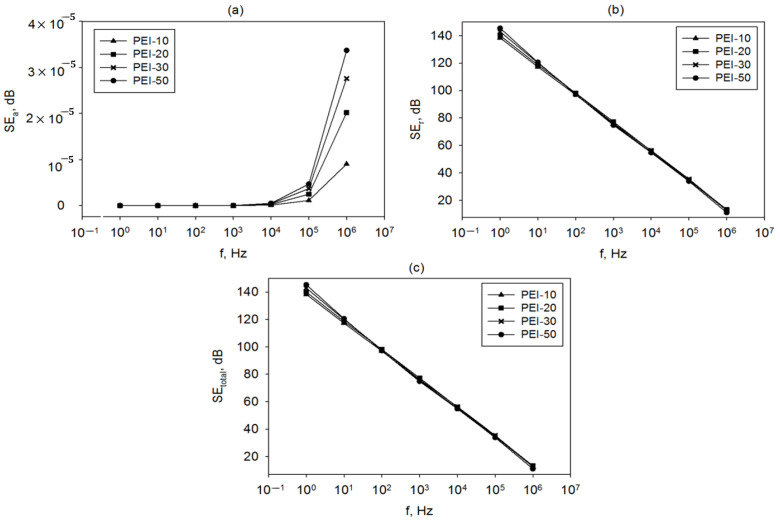
Shielding effectiveness by means of (**a**) absorption, (**b**) reflection, and (**c**) total shielding ability of the PEI/CoFe_2_O_4_ films.

The electric and magnetic properties of the materials are important for determining if they are able to cut off or attenuate the EMR produced by high-frequency sources. This can be achieved by producing reflection and absorption of the incident EMR onto the material surface. The theory of EMR compatibility states that the shielding phenomena noticed for far-field sources are similar for near-field sources, however, the sort of the source is essential for evaluating the effective shielding [63]. Thus, while absorption loss remains unaffected, the reflection loss is computed by replacing the wave impedance with the intrinsic impedance of the free space. This results in an asymptotic convergence when the distance between the source and the shielding layer is increased. The reflection factor for near-field electric sources becomes significantly larger than that of a uniform plane–wave source, and it is enhanced when the distance between the shielding material and source is reduced [63]. The electric field source was considered for the investigated PEI/CoFe_2_O_4_ nanocomposites. The attained shielding effectiveness data of the samples are depicted in Figure 10a,b. The graphs of the shielding from reflection and absorption of the EMR revealed an influence of the frequency. It can be remarked that when the frequency was higher, the EMR absorption loss tended to be higher, whereas the reflection was diminished. This is in agreement with the literature data for other polymer composites [64,65], which states that the magnitude of conductivity and permeability is extremely important such that reflection effectiveness is impacted by the ratio *σ/μ* and the absorption is influenced by their product [66]. The deviation of the waves via reflection by the PEI/CoFe_2_O_4_ films is caused by the distinct impedance of the shielding layer and the surrounding free space, while EMR attenuation occurs when unreflected waves penetrate the interior of the nanocomposite and is slightly stopped by dielectric and magnetic characteristics. Analysis of the results from Figure 10 reveals the dominant role of the reflection in regard to the absorption in effectively shielding the EMR by the PEI/CoFe_2_O_4_ samples. By the addition of the values corresponding to the reflection and absorption shielding effectiveness, it seems that the overall effectiveness (Figure 10c) ranges at 1Hz from 138.34 dB (10 wt% filler) to 145.41 dB (50 wt% filler), while at 1 MHz is around 11.02 dB (10–50 wt% filler). Therefore, the incorporation of the CoFe_2_O_4_ nanoparticles in the matrix determines a slight enhancement of the shielding effectiveness. The obtained data for overall shielding effectiveness were larger than the values reported in other articles for polymer composites, such as ZIF-67/wood [67] and silver nanowire/cellulose [68], while our results were smaller than that reported for materials based on carbon nanotubes/aramid nanofibers [69]. The literature [70] has formulated a classification of the EMI shielding materials: (a) low-level shielding layers (10–30 dB), (b) medium-level shielding layers (30–60 dB), (c) high-level shielding layers (60–90 dB), and (d) high-precision shielding layers (>90 dB). Hence, it seems that the prepared composites were fitted at 1 Hz as low-level shielding films suitable for low-end shielding uses. At 1 kHz, the PEI-based composites presented the shielding requirements of the military industry and aerospace domains. On the other hand, at 1 MHz the samples could be regarded as high-precision shielding films, which fulfill the demands for high-sensitive precision electronic products.

## 3. Materials and Methods

### 3.1. Raw Materials

The 4,4′-(4,4′-Isopropylidenediphenoxy)bis(phthalic anhydride) (6HDA) was provided from Aldrich and used as received. 2,6-Bis(3-aminophenoxy)benzonitrile (BDA) was synthesized following a method previously reported [71]. Cobalt ferrite nanoparticles were prepared by a co-precipitation method using carboxymethylcellulose as the surfactant, as previously described [72]. The average crystallite size, determined from the X-ray diffraction pattern, was found to be 21 nm. The saturation and remanent magnetization of pure cobalt ferrite was 50 emu g^−1^ and 22 emu g^−1^, respectively. The samples presented a high coercive field of 760 Oe.

### 3.2. Preparation of Nanocomposite Films

**Scheme 1:**
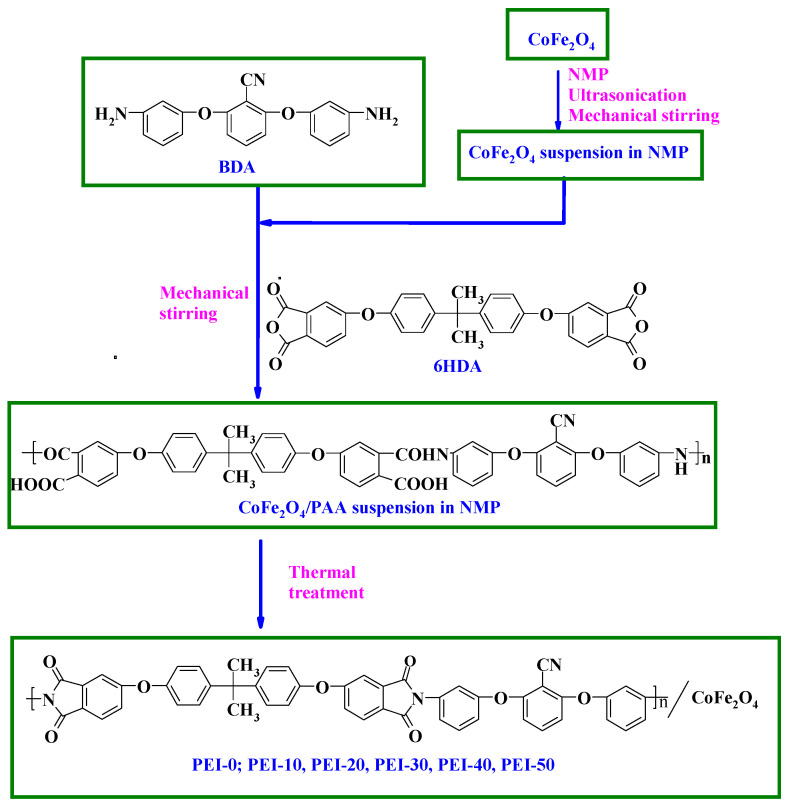
Preparation of magnetic polyetherimide nanocomposite films containing cobalt ferrite.

The polyetherimide nanocomposite films were prepared by incorporating cobalt ferrite nanoparticles in a poly(amic acid) solution, followed by film casting and thermal imidiaztion, as shown in Scheme 1.

Cobalt ferrite nanoparticles and N-methyl-2-pyrrolidone (NMP) (11 mL) were introduced into a flask equipped with a mechanical stirrer and a nitrogen inlet and outlet. The resulting mixture was sonicated for 2 h, and then stirred for 6 h to obtain a suspension. Afterward, BDA (0.634 g, 0.002 mol) was added, and the mixture was stirred under nitrogen to complete the dissolution of the diamine. 6HDA (1.04 g, 0.002 mol) was introduced and stirring was continued at room temperature for 12 h. Entrapped air bubbles were removed under vacuum. The resulting suspension was cast onto glass plates and heated at 50, 100, 150, and 200 °C for 1 h each and at 250 °C for 2 h. The inorganic content of the nanocomposites was controlled by the portion ratio between ferrite and poly(amic acid). The theoretical cobalt ferrite content of PEI-0, PEI-10, PEI-20, PEI-30, PEI-40, and PEI-50 was 0, 10, 20, 30, and 50 wt%, respectively. The thickness of the films was around 0.15 mm.

### 3.3. Methods

Infrared spectra were collected on a Bruker Vertex 70 Spectrometer (Bruker, Bremen, Germany) in the frequency window of 400 to 4000 cm^−1^ on KBr pellets or films. 

A scanning electron microscope (SEM) model Quanta 200 (Field Electron and Ion Company, Hillsboro, OR, USA)was employed for the morphological examination of the films (10 kV). For this purpose, the PEI and nanocomposites were fractured, and the cross-section profile was investigated. The energy-dispersive X-ray (EDX) component was used to achieve the elemental mapping.

Thermogravimetric (TG) and thermogravimetric derivative (DTG) data were attained on the Mettler Toledo TGA-SDTA851e (Mettler-Toledo International Inc., OH, USA) instrument operating in an N_2_ environment by setting a flow rate of 20 mL min^−1^ and a heating rate of 10 °C min^−1^. During the experiments, the temperature was varied between 25–900 °C, and the used sample weight ranged from 2.6 to 4.7 mg. 

Differential scanning calorimetry (DSC) data were registered on the Mettler Toledo DSC1 instrument (Mettler-Toledo International Inc., OH, USA) operating in an inert environment. The heating rate was fixed at 10 °C min^−1^ and the nitrogen purge was 100 mL min^−1^. During the scans, the temperature was swept from 25 °C to 350 °C. The mass of PEI and nanocomposites was between 2.4 and 4.7 mg. The data was processed using STARe software v.9.

The magnetic characteristics were recorded on a vibrating magnetometer (AGM and VSM Magnetometer, Princeton Measurement Co., NJ, USA) in ambient conditions.

A broadband dielectric spectrometer (Novocontrol Technologies, Montabaur, Germany) was employed for dielectric characterization. The alpha-A high-performance frequency analyzer was utilized for alternating electric field oscillations in the domain of 1–10^6^ Hz. The Quatro Cryosystem unit allowed temperature variation from −150 °C to 250 °C. The samples were interfaced between the electrodes, and the tests were performed under a dry N_2_ environment.

For overlapped dielectric signals, each *ε″*(*f*) dependency was fitted accordingly with Havriliak–Negami (*HN*) expression (1):

(1) $$\varepsilon^{*}=\varepsilon^{\prime }-i\varepsilon^{\prime \prime }=\varepsilon_{u}+\frac{\varepsilon_{r}-\varepsilon_{u}}{{\left[1+{\left(i\omega \tau_{HN}\right)}^{\alpha_{HN}}\right]}^{\beta_{HN}}}$$

where *ε_r_* and *ε_u_* are the relaxed (*ω*→0) and unrelaxed (*ω*→∞) values of the dielectric constant, *ω* = 2π*f* is the angular frequency, *f* is the frequency of the alternating field, *τ_HN_* represents the HN relaxation time of the process, α_HN_ and *β_HN_* describe the width and the asymmetry of the dielectric loss peak [73]. The HN fitting was performed with the WinFit software package provided by Novocontrol. The experimental data were separated with HN functions assigned with characteristic relaxation process types, and an additional exponential term for the contribution of the conductivity signal (*kω*^−*n*^; where *k* = *σ*_0_/*ω*_0_, *σ*_0_, and *ω*_0_ are the conductivity and permittivity of free space, respectively, and *n* represents the exponential fitting parameter). Furthermore, the relaxation time of the dielectric peak maxima, *τ_max_*, was estimated from *τ_HN_* parameter, considering the expression (2):

(2) $$\tau_{max}=\tau_{HN}{\left[\frac{sin\frac{\pi ab}{2+2b}}{sin\frac{\pi a}{2+2b}}\right]}^{\frac{1}{a}}$$

The segmental *α*-relaxation was described adequately with the Vogel–Fulcher–Tammann (VFT) Equation (3):

(3) $$\tau_{max}\left(T\right)=A\exp\left(\frac{B}{T-T_{V}}\right)$$

where *A* (s) is the relaxation time at infinite temperature, *B* (*K*, equivalent temperature) is the apparent activation energy of the process, and *T_V_* (K) is the Vogel temperature, expressing the deviation from linearity of the *α*-relaxation process [74].

## 4. Conclusions

This work aimed to develop novel PEI/CoFe_2_O_4_ materials as EMI shielding layers. The nanocomposite films were developed by incorporating nano-sized cobalt ferrite particles into a polyamic acid solution, followed by film casting and controlled heating to achieve the corresponding imide structure. Nanocomposite films with 10, 20, 30, 40, and 50 wt% content of ferrite particles are compared with the neat PEI film. The influence of filler content on the film properties was evidenced. SEM investigations showed an increase in particle agglomeration at high ferrite content. According to TGA analysis, the introduction of ferrite leads to a decrease in decomposition temperature from 485 °C to 450 °C when 50 wt% particles are inserted. The saturation and remanent magnetization values are in the range of 4.39–27.9 and 1.47–9.57 emu g^−1^, respectively, and can be easily assigned to mass content in the samples while the coercivity remains almost constant at the value corresponding to pure ferrite nanoparticles. The dielectric properties determined by broadband dielectric spectroscopy measurements are strongly related to the cobalt ferrite loading levels. The neat film exhibits a low dielectric constant, which increases at high magnetic filler content in the PEI-based composite. The dependence of dielectric loss on temperature reveals two subglass relaxations, *γ* and *β*, and a primary *α*-relaxation at high temperatures for the films containing up to 20 wt% particles. By introducing magnetic nano inclusions, the conductivity increases considerably, especially for the samples with 30 and 50 wt% filler content. The shielding effectiveness is dominated by the reflection of EMR and less influenced by the incident wave absorption. The shielding efficiency is influenced by the frequency, revealing that the EMI shielding ability of the PEI/CoFe_2_O_4_ materials renders wide applicability ranging from low-end shielding uses to high-sensitive precision electronic products.

## Figures and Tables

**Table 1:** The main thermal characteristics of the PEI-0, PEI-10, PEI-30, and PEI-50.

Sample	Stage	*T_onset_* (°C)	*T_peak_* (°C)	*T_endset_* (°C)	*W* (%)	Residue (%)	*T_g_* (°C)
PEI-0	I	487	517	539	26.85	54.14	196
	II	539	585	704	19.01		
PEI-10	I	460	491	517	20.51	39.03	197
	II	517	551	630	15.19		
	III	682	697	-	25.27		
PEI-30	I	452	475	498	16.84	49.86	196
	II	498	576	596	10.74		
	III	658	681	798	22.56		
PEI-50	I	450	470	493	12.35	50.58	196
	II	493	572	586	9.21		
	III	665	689	703	27.86		

**Table 2:** Magnetic parameters *Ms*, *Mr*, and *Hc* of the PEI//CoFe_2_O_4_ films.

Sample	*Ms* (emu g^−1^)	*Mr* (emu g^−1^)	*Hc* (Oe)
PEI-10	4.39	1.47	759
PEI-20	9.91	3.59	757
PEI-30	15.6	4.89	736
PEI-40	17.9	5.58	765
PEI-50	27.9	9.57	705

**Table 3:** Parameters of the VFT equation for the segmental α-relaxation.

Sample	*A* (s)	*T_V_* (K)	*B* (K)
PEI-0	6 × 10^−12^	427	1194
PEI-10	2 × 10^−12^	418	1412
PEI-20	3 × 10^−13^	402	2005

**Table 4:** Numerical values of the measured conductivity derived at 1 Hz and temperatures around room temperature (T = 25 °C), below *T_g_* (T = 150 °C), and above *T_g_* (T = 250 °C) for the PEI and PEI/CoFe_2_O_4_ films.

Sample	Measured Conductivity, *σ_m_* (S/cm)		
	T = 25 °C	T = 150 °C	T = 250 °C
PEI-0	1.6 × 10^−14^	8.6 × 10^−14^	1.1 × 10^−9^
PEI-10	4.7 × 10^−14^	1.1 × 10^−13^	7.1 × 10^−10^
PEI-20	8.7 × 10^−14^	4.4 × 10^−13^	3.8 × 10^−10^
PEI-30	2.0 × 10^−13^	3.0 × 10^−11^	6.2 × 10^−8^
PEI-50	4.7 × 10^−13^	2.9 × 10^−10^	1.1 × 10^−6^

## Data Availability

Data is contained within the article or Supplementary Material.

## Supplementary Materials

The following supporting information can be downloaded at: https://www.mdpi.com/article/10.3390/ijms24020999/s1.
