# Supplementary material for: Cobalt Ferrite/Polyetherimide Composites as Thermally Stable Materials for Electromagnetic Interference Shielding Uses
Source: Int J Mol Sci. 2023 Jan 5;24(2):999. doi: 10.3390/ijms24020999 (PMC9864334; doi:10.3390/ijms24020999)
Supplement: Supplementary file 1 [file ijms-24-00999-s001.zip › ijms-2109096-supplementary.pdf]

Cobalt Ferrite/Polyetherimide Composites as Thermally Stable Materials for Electromagnetic Interference Shielding Uses

Mihai Asandulesa [1], Corneliu Hamciuc [1], Aurel Pui [2], Constantin Virlan [2], Gabriela Lisa [3], Andreea Irina Barzic [1,*] and Bogdan Oprisan [4]

[1] "Petru Poni" Institute of Macromolecular Chemistry, 41A Grigore Ghica Voda Alley, 700487 Iasi, Romania

[2] Faculty of Chemistry, "Alexandru Ioan Cuza" University, 700506 Iasi, Romania

[3] Faculty of Chemical Engineering and Environmental Protection "Cristofor Simionescu",

"Gheorghe Asachi" Technical University of Iasi-Romania, 73 Prof. dr. doc. D. Mangeron Street, 700050 Iasi, Romania

[4] Faculty of Medicine, Discipline Biophysics and Medical Physics, "Grigore T. Popa" University of Medicine and Pharmacy, 16 University Str., 700115 Iasi, Romania

* Correspondence: irina_cosutchi@yahoo.com

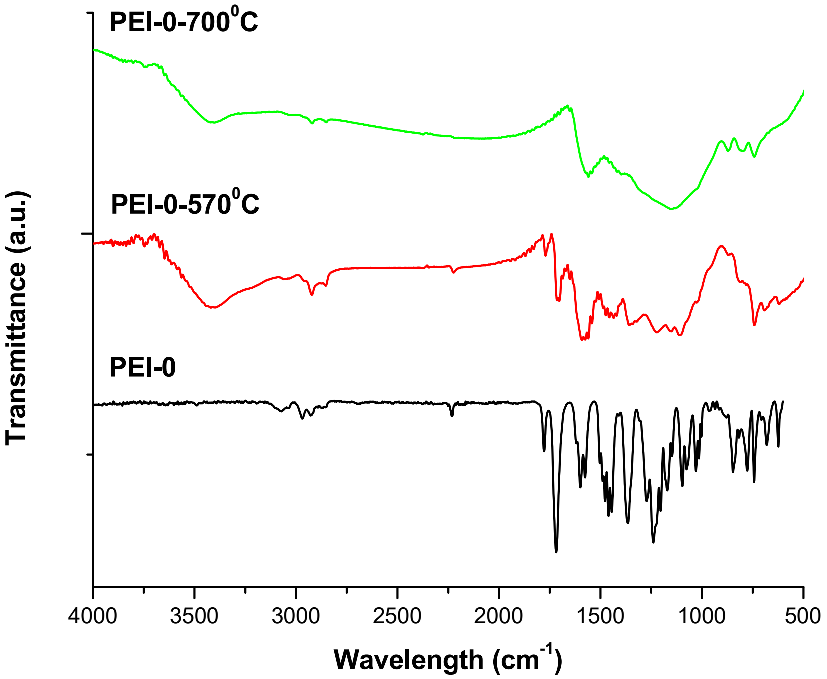

Figure S1. FTIR spectra of PEI-0 and of PEI-0 heated up to 570 and 700°C, with the heating rate of 10°C min^{-1}, in air atmosphere.

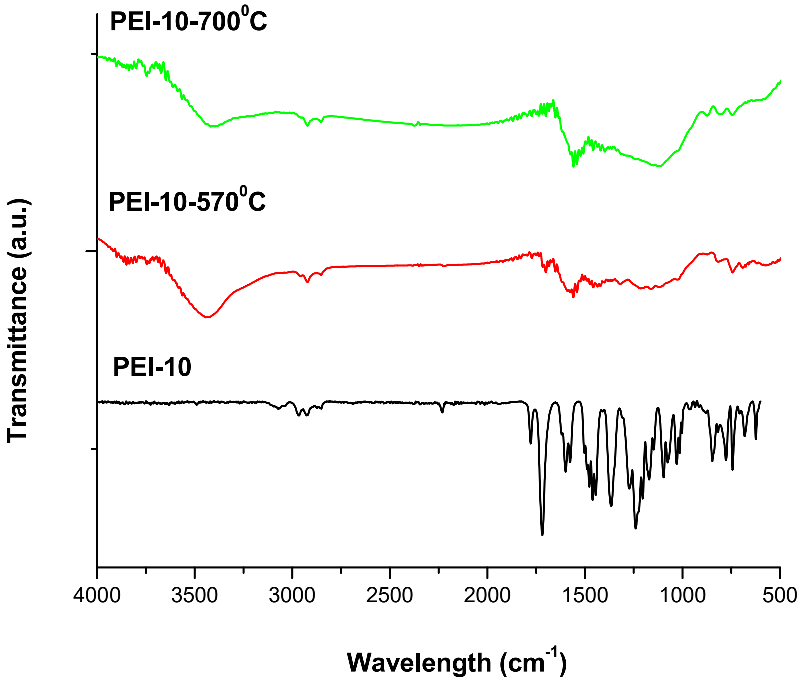

Figure S2. IR spectra of PEI-10 and of PEI-10 heated up to 570 and 700°C, with the heating rate of 10°C min-1, in air atmosphere.

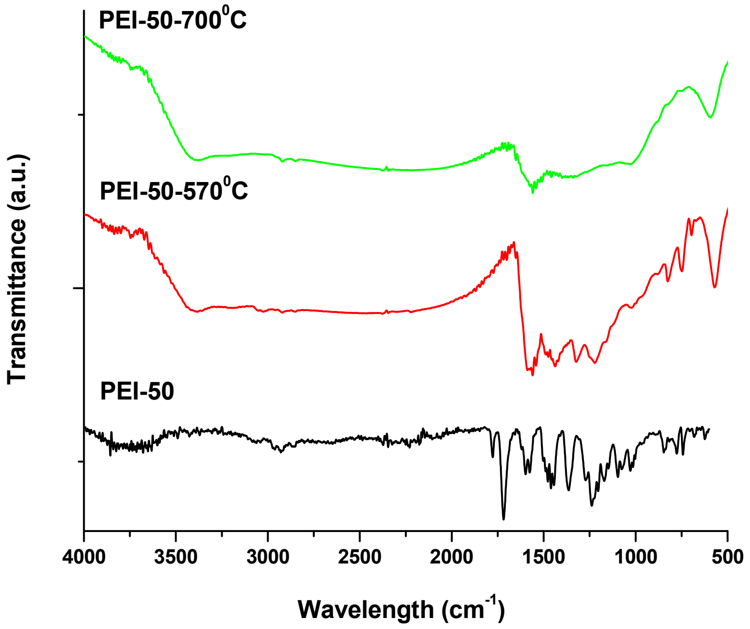

Figure S3. IR spectra of PEI-50 and of PI-50 heated up to 570 and 700°C, with the heating rate of 10°C min-1, in air atmosphere.

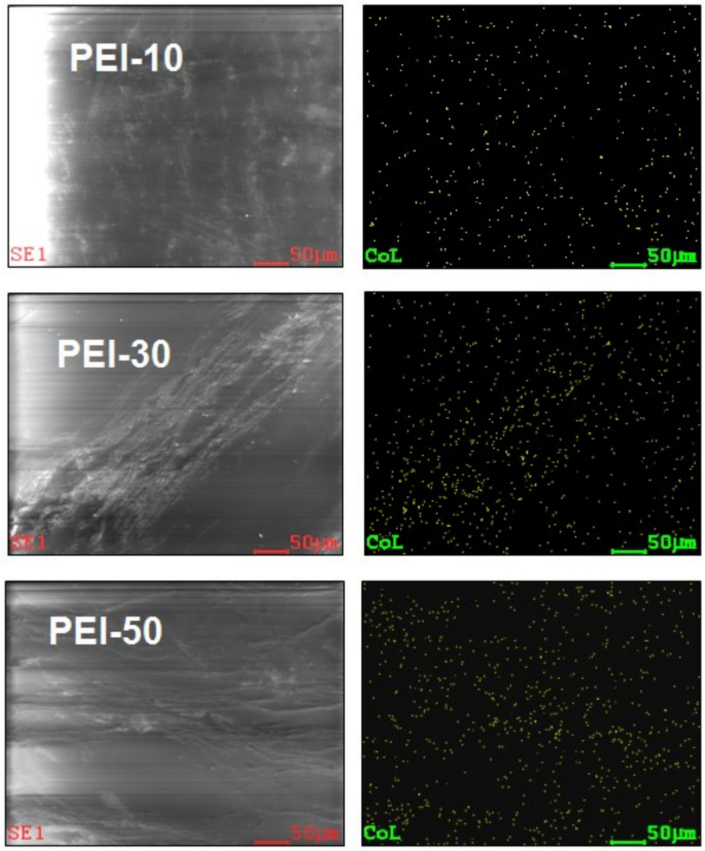

Figure S4. EDX mapping of hybrid films PI-10, PI-30 and PI-50 (Co atoms).

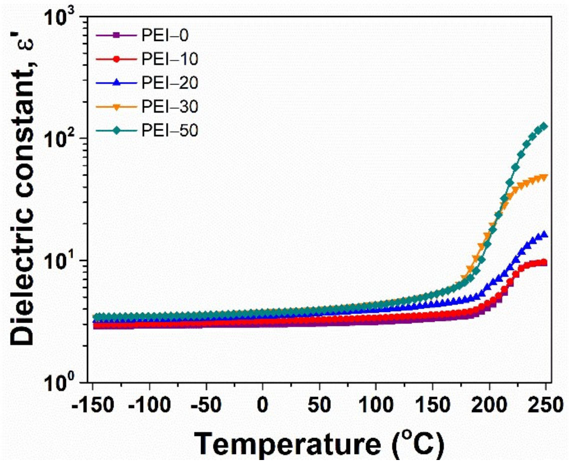

Figure S5. Temperature dependences of dielectric constant at 1 kHz for simple PEI-0 and nanocomposites PEI-10, PEI-20, PEI-30 and PEI-50.

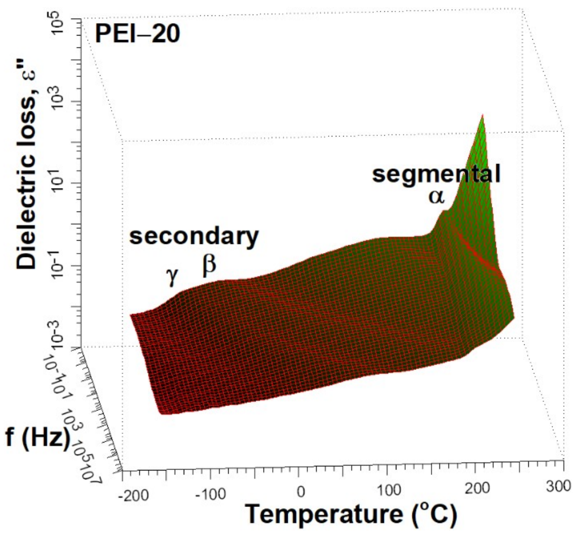

Figure S6. Evolution of dielectric loss with frequency and temperature for PEI-20 nanocomposite.
